# Stronger Association between Insomnia Symptoms and Shorter Telomere Length in Old HIV-Infected Patients Compared with Uninfected Individuals

**DOI:** 10.14336/AD.2018.0204

**Published:** 2018-12-04

**Authors:** Yingying Ding, Haijiang Lin, Sujuan Zhou, Keran Wang, Lingling Li, Yucheng Zhang, Yuan Yao, Meiyang Gao, Xing Liu, Na He

**Affiliations:** ^1^Department of Epidemiology, School of Public Health, Fudan University, Shanghai, China; ^2^The Key Laboratory of Public Health Safety of Ministry of Education, Fudan University, Shanghai, China; ^3^Taizhou City Center for Disease Control and Prevention, Taizhou City, Zhejiang, China

**Keywords:** HIV, older age, insomnia symptoms, telomere length

## Abstract

Growing evidence suggests that HIV infection may accelerate biological aging. Insomnia symptoms, particularly in later life, exacerbate cellular aging. We examined the association between insomnia symptoms and leukocyte telomere length (LTL), and further explored how this association was affected by HIV serostatus and age. Data were assessed from 244 HIV-infected individuals ≥40 years and 244 HIV-uninfected individuals who were frequency-matched by age, gender and education level. Insomnia symptoms were assessed by responses to four sleep-related questions covering the past month. We performed multivariable linear regression with logarithmically transformed LTL and reported exponentiated coefficients. HIV-infected individuals had shorter LTL compared to uninfected individuals (geometric mean 0.82 vs 0.89, *P=*0.052), and this association remained after adjustment for gender, education level, and smoking history (-7.4%, *P=*0.051) but markedly attenuated after additional adjustment for insomnia and depressive symptoms (-3.7%, *P=*0.367). Significant interactions between age group (55-82 vs 40-54 years) and insomnia symptoms on LTL were observed in the HIV-infected individuals (-28.4%, *P*=0.033) but not the uninfected (-17.9%, *P=*0.250)*.* After stratifying by age group, LTL was independently associated with insomnia symptoms in those 55 years and older among the HIV-infected individuals (-24.5%, *P=*0.026) but not those 40-54 years old (-9.8%, *P=*0.428). Our findings suggest that elevated insomnia and depressive symptoms may partly explain the correlation between HIV serostatus and shorter LTL. Significant association between insomnia and shorter LTL observed in elderly HIV-infected but not in uninfected individuals suggest that such adverse effect may begin at an earlier age or is more pronounced in HIV-infected individuals but requires further investigation.

Insomnia is characterized by difficulty in initiating and/or maintaining sleep [1]. Accumulating epidemiological evidence indicates that insomnia and sleep disorders are associated with a high risk of age-related conditions and all-cause mortality [2,3]. Potential pathways include promotion of biological processes that contribute to cellular aging, including cellular stress, accumulation of DNA damage, inflammation, and telomere shortening [4,5].

Telomeres are repetitive DNA sequences that are located at the ends of chromosomes and protect their integrity [6]. Telomere length (TL) in peripheral blood leukocytes or leukocyte TL (LTL) shortens with each cell division in most cells. There is increasing acceptance that telomere shortening is a biological marker for human aging and a possible mechanism underlying premature morbidity and mortality in humans [7,8]. The association between sleep quality and shorter LTL has been investigated in the general population, and those studies show positive associations of poor sleep quality, insomnia and short sleep duration with shorter LTL [9,10]. However, recent studies in the general population suggest that the effect of sleep loss is particularly pronounced in elderly adults [11,12], as they have reduced capacity to respond to physiological perturbations that place stress upon them [13].

The increased life expectancy of HIV-infected individuals due to combination antiretroviral therapy (cART) has revealed an increased incidence of age-related non-AIDS comorbidities [14]. The increase in age-related diseases and their premature onset suggest that they may be undergoing accelerated biological aging that is possibly mediated by increased cellular senescence [15-18]. Shorter LTL has been observed in HIV-infected patients compared with HIV-uninfected individuals [19,20]. A recent study suggests that acquisition of HIV itself and viral load are primarily responsible for the association between HIV infection and shorter LTL [20]. It is noteworthy that insomnia is common among HIV-infected adults [21-23], which may be attributed to HIV neurotoxicity, adverse effects of cART and, most consistently and importantly, depression [19-23]. Given the association of depression and insomnia with cellular aging observed in the general population [8-12,24], we speculate that insomnia symptoms may contribute to biological aging among HIV-positive individuals. We further speculate that such effects are likely more pronounced in the old HIV-infected individuals, as observed in the general population [11,12], and there are stronger effects of insomnia and age on shorter LTL (or beginning at an earlier age) due to stronger impaired stress responses and earlier age of onset among HIV-infected individuals [15,19,25].

Existing literature on the relationship between LTL and sleep parameters among HIV-infected patients is limited. To the best of our knowledge, only one study reported that LTL is associated with sleep duration but not with sleep quality among HIV-infected patients [26]. To explore these issues, we performed a cross-sectional study comparing HIV-infected and -uninfected individuals to investigate the association between insomnia symptoms and LTL serving as a biomarker of biological aging, and to further determine how this association is affected by HIV serostatus and age.

## MATERIALS AND METHODS

### Study participants

We conducted a cross-sectional study of HIV-infected and -uninfected individuals between June 2014 and May 2015 in Taizhou prefecture of Zhejiang province, China, as previously described [27,28]. All HIV-infected patients in China are required to register in the Comprehensive Response Information Management System (CRIMS) [29]. HIV-infected patients (≥40 years) in Taizhou registered with the CRIMS were consecutively enrolled. HIV-uninfected participants were recruited either from persons receiving HIV voluntary counseling and testing or routine physical examinations at the local Center for Disease Prevention and Control during the study period. For this analysis, 244 HIV-infected and 244 -uninfected individuals were randomly selected according to the following criteria: 1) sufficient volume of blood samples for LTL measurements, 2) both groups were frequency matched in a 1:1 ratio by gender, education level, and 5-year age categories.

The study was approved by the Institutional Review Board of Fudan University, Shanghai, China. All subjects provided informed consent prior to enrollment.

### Data Collection 

Participants underwent standardized screening for demographics, insomnia and depressive symptoms, and a physical examination of height, weight, waist and hip circumference. The cut-off of waist to hip ratio (WHR) for abdominal obesity was 0.9 for men and 0.85 for women [30]. HIV- and treatment-related information was extracted from CRIMS records.

### Insomnia and Depressive Symptom Measurements

Insomnia symptoms were assessed with responses to four sleep-related questions based on the work by Jenkins Sleep Problems Scale [31]. All items corresponding to the nighttime insomnia symptoms specified by the Diagnostic and Statistical Manual of Mental Disorders, Fourth Edition (DSM-IV). These were: “Over the last month did you: have trouble staying asleep, have trouble falling asleep, wake up too early and feel unable to get back to sleep, or wake up several times per night and feel unable to get back to sleep?” There were four possible responses to each question: “not at all”, “some nights”, “most nights” and “every night”. Our definition of insomnia symptoms was based on answers with “most nights” or “every night” to one or more questions. Depressive symptoms were measured by the 10-item adapted version of Zung Self-Rating Depression Scale (SDS) with higher scores indicating a greater degree of depression (range: 0-40) [32,33].

**Table 1:** Sample characteristics of HIV-infected and uninfected participants stratified by age group.

Characteristics	HIV-infected participants				HIV-uninfected participants				
	
	All	40-54 years	55-82 years	*P*^a^	All	40-54 years	55-82 years	*P^a^*	*P^a^*
*Subjects*	244	157	87		244	157	87		
Age, years	52.4±9.0	46.7±36.5	63.0±6.3	<.001	52.5±9.1	46.7±3.7	62.9±6.0	<.001	0.933
Male	180 (73.8)	118 (75.2)	62 (71.3)	0.508	180 (73.8)	118 (75.2)	62 (71.3)	0.508	1.000
Junior middle school or obove	145 (59.4)	115 (73.2)	30 (34.5)	<.001	145 (59.4)	115 (73.2)	30 (34.5)	<.001	1.000
BMI, kg/m^2^	22.1 (20.3-24.0)	22.4 (20.6-24.3)	21.5 (19.9-23.1)	0.002	23.7 (22.1-26.0)	24.2 (22.3-26.5)	23.4 (21.5-25.4)	0.028	<.001
Waist circumference, cm	81.0 (75.5-86.0)	81.0 (75.0-86.0)	81.0 (76.0-88.0)	0.118	84.0 (78.0-90.0)	84.0 (78.0-89.0)	86.0 (80.0-91.0)	0.070	<.001
Hip circumference, cm	90.0 (84.7-96.0)	90.0 (85.5-96.0)	88.7 (84.0-95.0)	0.104	95.0 (90.0-99.0)	96.0 (90.0-100.0)	94.0 (90.0-98.0)	0.249	<.001
WHR above the cutoff	154 (63.1)	96 (61.1)	58 (66.7)	0.392	133 (54.5)	75 (47.8)	58 (66.7)	0.004	0.053
Smoking history				0.119				0.115	0.021
Current smoker	70 (28.0)	49 (31.2)	21 (24.1)		94 (37.6)	58 (36.9)	36 (41.4)		
Previous smoker	29 (11.6)	14 (8.9)	15 (17.2)		16 (6.4)	7 (4.5)	9 (10.3)		
Never smoked	145 (59.4)	94 (59.9)	51 (58.6)		134 (54.9)	92 (58.6)	42 (48.3)		
Current alcohol user	18 (7.4)	11 (7.0)	7 (8.0)	0.766	20 (8.3)	9 (5.7)	11 (12.6)	0.059	0.735
Depressive symptoms as a continuous variable	16.6±4.8	16.5±4.9	16.6±4.7	0.901	13.3±3.7	13.4±3.8	16.4±4.9	0.039	<.001
Insomnia symptoms	46 (18.9)	28 (17.8)	18 (20.7)	0.585	23 (9.4)	12 (7.6)	11 (12.6)	0.200	0.003
Insomnia symptoms as a continuous variable	6.5±2.7	6.5±2.6	6.6±2.9	0.894	5.8±2.2	5.5±1.9	6.4±2.5	0.003	0.002
*HIV-related parameters*									
Homosexual HIV transmission	45 (18.4)	36 (22.9)	9 (10.3)	0.015					
Years since HIV diagnosis	3.0 (1.6-4.7)	2.7 (1.5-4.6)	3.5 (2.0-4.7)	0.187					
Nadir CD4 count, cells/μL				0.394					
< 100	60 (24.5)	43 (27.4)	17 (19.5)						
100-199	87 (35.7)	54 (34.4)	33 (37.9)						
≥ 200	97 (39.7)	60 (38.2)	37 (42.5)						
Current CD4 count ≥ 200 cells/μL	205 (84.0)	132 (84.1)	79 (83.9)	0.973					
Using cART at enrollment				0.277					
cART naïve	20 (8.2)	16 (10.2)	4 (4.6)						
Duration on cART < 3 years	133 (54.5)	82 (52.2)	51 (58.6)						
Duration on cART ≥ 3 years	91 (37.3)	59 (37.6)	32 (36.8)						
Duration on cART^b^, years	2.4 (0.9-3.7)	2.0 (0.9-3.7)	2.6 (0.8-3.8)	0.544					
Using EFV at enrollment	127 (52.0)	80 (51.0)	47 (54.0)	0.646					
Plasma HIV RNA < 200 copies/mL^c^	158 (90.3)	102 (92.7)	56 (86.1)	0.156					
Leukocyte telomere length (LTL)	0.91±0.42	0.92±0.40	0.89±0.44	0.657	0.96±0.40	0.97±0.39	0.94±0.41	0.590	0.178
Geometric LTL^d^	0.82 (0.77-0.87)	0.83 (0.77-0.89)	0.80 (0.72-0.88)	0.548	0.89 (0.84-0.93)	0.90 (0.84-0.95)	0.86 (0.79-0.94)	0.480	0.052

Data are no. (%), mean±SD or median (interquartile range [IQR]), unless otherwise indicated.

a By the χ^2^, Fisher’s exact test or student’s t-test, Wilcoxon rank-sum test, as appropriate.

b Data are available for 224 HIV-infected cART recipients.

c Data are available for 175 HIV-infected participants.

d Refers to geometric mean and 95% confidence interval. BMI, body mass index; cART, combination antiretroviral therapy; WHR, waist to hip circumference ratio.

### LTL Measurement

At enrollment, 5 mL of venous blood from each participant was collected into EDTA tubes. Whole blood was stored in 1.5 mL screw-cap Eppendorf tubes at -80 °C until analysis. Genomic DNA was extracted from peripheral blood leukocytes using the QIAampR Blood DNA Mini Kit (Qiagen, Germany) by standard procedures and stored at -20 °C for batch telomere length measurement. Genomic DNA was detected by 1% agarose gel electrophoresis. DNA concentration was calculated by measurement of 260nm OD value. Telomere length was measured by qPCR adapted from the published methods [34,35]. Primers tel-F: 5'-CGGTTTGTTTGGGTTTGGGTTTGGGTTTGGGTTTGGGTT-3' and tel-R: 5'-GGCTTGCCTTACCCTT ACCCTTACCCTTACCCTTACCCT-3' were used for amplification of telomere. Primers 36B4-F: 5'-AGCAAG TGGGAAGGTGTAATCC-3', and 36B4-R: 5'-CCCATT CTATCATCAACGGGTACAA-3' were used for amplification of the single copy gene (36B4). Both of the thermal cycling profiles were 30s at 95 ?, 40 cycles of 5 s at 95 ?, 34 s at 60 ?with signal acquisition. The 60 ? reads provided the Ct values for amplification of the telomere or 36B4-template. One reference human genomic DNA sample with four concentrations prepared in each 96-well plate was included in each PCR run to generate standard curves. The quantities of telometic products (T) and single-copy genes (S) were determined relative to the reference DNA by the standard curve method. The T/S ratio for each sample was measured twice. When the duplicate T/S value and the initial value varied by more than 15%, the sample was run for a third time, and the two closest values were then used to obtain the average TL. In addition, three control samples were included in each plate. If the inter-plate coefficient of variation (CVs) of control samples were less 15% and intra-plate CVs were less than 7%, the results were accepted. Otherwise, the samples were re-tested until they were within the range.

### Statistical Analyses

All statistical analyses were performed using SAS software (version 9.11). Age was dichotomized into two groups: 40-54 years old and 55-82 years old. Data on LTL were log-transformed so that the values were approximately normally distributed. Differences were assessed using Chi-square test, Fisher’s exact test or student’s t-test, and/or Wilcoxon rank-sum test, as appropriate.

We performed univariable and multivariable linear regression models with log-transformed LTL as a continuous variable. Obtained coefficients were exponentiated to obtain the differences in LTL on a multiplicative scale, which could be interpreted as the percent differences in LTL. Variables considered included age, gender, education, smoking history, WHR above cutoff, insomnia and depressive symptoms, nadir and current CD4 count, cART status, HIV RNA <200 copies/mL. Variables with *P* value <0.15 in univariable analysis were used as candidates for development of a multivariable model. We first explored whether HIV serostatus was independently associated with shorter LTL. Interaction term between age groups and insomnia symptoms was examined in all participants. We next examined the factors associated with LTL and interaction effects of insomnia symptoms and age group in the HIV-infected and -uninfected individuals. In an additional analysis, we repeated the analysis in both HIV-infected and -uninfected groups stratifying by age group, mainly to check how the association of insomnia and LTL was affected by age group and HIV serostatus; and age as a continuous variable was a *priori* included in these final models because it is an established risk factor for shorter LTL. Depression has been shown to be associated with shorter LTL [34], and it is also highly correlated with insomnia symptoms in HIV-infected people as well as in the general population [21,22]; to avoid collinearity between them, each of the variables was included without the another in two separate models (multivariate models 1 and 2). In addition, to test the robustness of the association between insomnia and shorter LTL, sensitivity analysis was performed when treating insomnia symptoms as a continuous variable.

## RESULTS

### Sample Characteristics

The HIV-infected and uninfected groups were comparable in age (median, approx. 50.0 years), gender (73.8% male) and education level (59.4% middle school or higher). HIV-infected participants more frequently had WHR above cutoff, had previously been smokers, and had a higher level of depressive symptoms, but were less likely to be current smokers and/or have lower average body mass index (BMI, weight in kilograms/height in square meter) and waist circumference. Nearly all HIV-infected participants (94.4%) were receiving cART (Table 1).

### Insomnia Symptoms 

Overall, HIV-infected participants had a higher prevalence of insomnia symptoms than uninfected participants (18.9% vs 9.4%, *P=*0.032). After stratifying by age group, the prevalence of insomnia symptoms was significantly higher in HIV-infected than HIV-uninfected participants in the middle age group (17.8% vs 7.6%, *P*=0.007), but such difference was not significant in the aged group (20.7% vs 12.6%, *P*=0.154) (Table 1). In multivariable Logistic regression analysis, insomnia symptoms were significantly associated with only depressive symptoms in all HIV-infected participants and were either marginally significantly or significantly associated with age, being female, and depressive symptoms in all HIV-uninfected participants (Supplementary Table 1).

**Table 2:** Multivariable analyses of the association between various factors and leukocyte telomere length in HIV-infected participants according to age group.

	Percentage change (95% CI)^a^	*P*
All HIV-infected participants		
Age group (55-82 vs 40-54 years)	3.8 (-0.8 to 17.3)	0.668
Insomnia symptoms	8.5 (-10.2 to 31.1)	0.397
Current CD4 count ≥ 200 cells/μL	15.9 (-1.1 to 35.8)	0.069
Insomnia symptoms × age group interaction	-28.4 (-2.8 to 47.2)	0.033
HIV-infected participants aged 40-54 years		
Age as a continuous variable	-8.0 (-9.8 to -6.1)	0.412
Insomnia symptoms	9.7 (-10.0 to 31.5)	0.397
Current CD4 count ≥ 200 cells/μL	20.0 (-1.7 to 46.6)	0.076
HIV-infected participants aged 55-82 years		
Model 1^b^		
Age as a continuous variable	-1.2 (-2.9 to 0.4)	0.108
Insomnia symptoms	-24.5 (-40.8 to -3.7)	0.026
Model 2^b^		
Age as a continuous variable	-0.9 (-2.5 to 0.7)	0.267
Depressive symptoms as a continuous variable	-2.5 (-4.5 to -0.5)	0.018

a Other variables considered in model selection included age, gender, insomnia symptoms, depressive symptoms, nadir CD4 count, cART status, HIV RNA <200 copies/mL which were selected by univariable linear regression models with *P *<0.15. Backward selection was used with retention at *P *<0.10. Age and insomnia symptoms were enforced and included in the final model where appropriate. Only variables included in final model were presented.

b When both insomnia and depressive symptom were simultaneously included in the final model, they were no more significant (*P*=0.186 and *P*=0.119, respectively). CI, confidence interval; WHR, waist to hip circumference ratio.

### Leukocyte Telomere Length and its Association with HIV infection

Overall, HIV-infected participants had shorter LTL than uninfected participants (geometric mean 0.82 vs 0.89, *P*=0.052) (Table 1). In multivariable analysis adjusting for gender, education level, and smoking history, HIV infection was associated with shorter LTL (-7.4%, 95% CI -14.4% to 0.0%, *P*=0.051). This association were markedly attenuated after additional adjustment for insomnia and depressive symptoms (-3.7%, 95% CI -11.3% to 4.5%, *P*=0.367). No interactions with HIV serostatus on LTL were detected.

To check whether the association between HIV serostatus and LTL exists for patients with viral load suppression on cART, we further conducted a subgroup analysis limiting to HIV-infected participants receiving cART and who had HIV RNA <200 copies/mL (*n*=158) and all uninfected individuals. HIV infection was independently associated with shorter LTL (-10.4%, 95% CI -17.7% to -2.4%, *P*=0.012) after adjustment for age, gender, education level, and smoking history. The association was attenuated (-6.5%, 95% CI -14.4% to 2.2%, *P*=0.139) after additional adjustment for insomnia and depressive symptoms. Of note, HIV-infected subjects with available plasma HIV RNA data had more nadir CD4 count <100 cells/μL, longer period since HIV diagnosis, and longer treatment duration than those without such data (Supplementary Table 2).

### Main and Interaction Effects of Age group and Insomnia Symptoms on LTL by HIV serostatus and among all participants 

**Figure 1.**
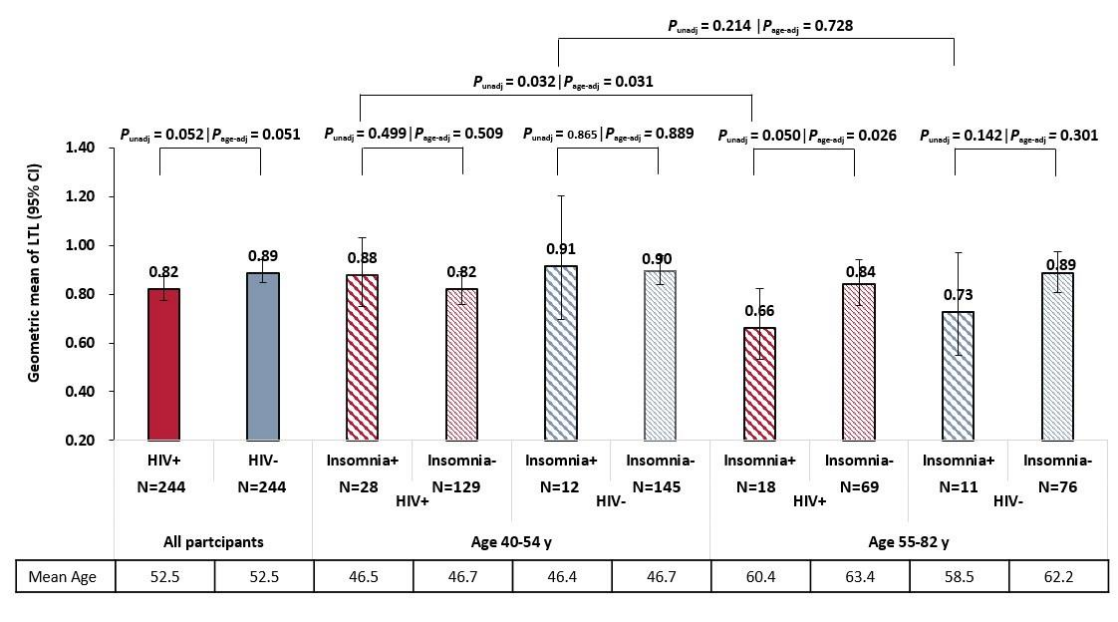
Geometric mean and 95% confidence interval of leukocyte telomere length in all HIV-infected and HIV-uninfected participants and according to age group. *P*_unadj _was calculated using t-test, *P*_adj _was calculated using multivariable linear regression model adjusting for age as continuous variable. Abbreviations: age-adj, age adjusted; CI, confidence interval; LTL, leukocyte telomere length; unadj, unadjusted.

The geometric means of LTL stratified by HIV serostatus and age group are shown in Figure 1. We noted that among the aged HIV-infected participants (55-82 years), those with insomnia symptoms had lower geometric means of LTL than those without insomnia symptoms (0.664 vs 0.84; *P*_unadjusted_*=*0.050 and *P*_age-adjusted_=0.026). A similar trend was observed in the aged HIV-uninfected participants but did not reach significance (0.73 vs 0.89; *P*_unadjusted_=0.142 and *P*_age-adjusted_=0.301). In contrast, among both HIV-infected and uninfected participants who were 40-54 years old, LTL was similar regardless of insomnia symptoms (Fig. 1).

Among all participants, there was a significant interaction between age group and insomnia symptoms (-25.0%, 95% CI -40.0% to -6.1%, *P*_interaction_=0.012) on LTL after adjusting for HIV serostatus, gender, education level, smoking history, and depressive symptoms. No main effects of age group (55-82 vs 40-54 years) and insomnia symptoms on LTL were identified. We repeated the analysis of HIV-infected and HIV-uninfected participants, and found that in multivariate models, the interaction between age group and insomnia symptoms was significant (*P*_interaction_=0.033) among HIV-infected participants (Table 2) but not the HIV-uninfected participants (*P*_interaction_=0.250) (Table 3).

### Other Determinants of LTL by HIV serostatus and Age Group

Univariable analyses of the associations between various factors and LTL in all participants according to HIV serostatus and age groups are shown in Supplementary Table 3. In multivariable models LTL was only marginally significantly associated with a current CD4 count of <200 cells/μL (*P*=0.069) among all HIV-infected participants in addition to the significant interaction between age and insomnia symptoms as described above (Table 2), whereas LTL was significantly associated with at least a junior middle school education level and depressive symptoms among all HIV-uninfected participants (Table 3).

We repeated the analysis after further stratification by age group. In multivariable models, only current CD4 count of <200 cells/μL (*P*=0.076) was marginally significantly associated with shorter LTL among the middle-aged HIV-infected participants, whereas depressive symptoms (*P*=0.026) and insomnia symptoms (*P*=0.018) were significantly associated with shorter LTL among the old HIV-infected participants when one was included without the other (Table 2). Among the HIV-uninfected participants, age (*P*=0.064) and depressive symptoms (*P*=0.006) were either marginally significantly or significantly associated with shorter LTL among the middle-aged HIV-uninfected participants, whereas having at least junior middle school education level (*P*<0.001), WHR above cutoff (*P*=0.006), and depressive symptoms (*P*=0.090) were significantly or marginally significantly associated with shorter LTL among the old HIV-uninfected participants (Table 3).

**Table 3:** Multivariable analyses of the association between various factors and leukocyte telomere length in HIV-uninfected participants according to age group.

	Percentage change (95% CI)^a^	*P*
All HIV-uninfected participants		
Age group (55-82 vs 40-54 years)	-4.9 (-15.2 to 6.7)	0.396
Junior middle school or above	-14.0 (-22.8 to -4.3)	0.006
Depressive symptoms as a continuous variable	-2.5 (-3.8 to -1.2)	<.001
Insomnia symptoms	8.4 (-13.9 to 36.5)	0.492
Insomnia symptoms × age group interaction	-17.9 (-41.3 to 14.9)	0.250
HIV-uninfected participants aged 40-54 years		
Age as a continuous variable	-1.6 (3.3 to 0.1)	0.064
Depressive symptoms as a continuous variable	-2.4 (-4.0 to -0.7)	0.006
Insomnia symptoms	7.3 (-14.8 to 35.0)	0.550
HIV-uninfected participants aged 55-82 years		
Model 1^b^		
Age as a continuous variable	-0.9 (-2.2 to 0.4)	0.195
Junior middle school or above	-29.9 (-41.0 to -16.7)	<.001
WHR above the cutoff	-20.7 (-33.6 to -5.3)	0.012
Insomnia symptoms	-9.8 (-30.0 to 16.2)	0.428
Model 2^b^		
Age as a continuous variable	-0.7 (-2.0 to 0.7)	0.314
Junior middle school or above	-27.9 (-39.4 to -14.3)	<.001
WHR above the cutoff	-22.1 (-34.5 to -7.0)	0.006
Depressive symptoms as a continuous variable	-2.0 (-4.2 to 0.3)	0.090

a Other variables considered in model selection included age, gender, education, smoking history, WHR above the cutoff, insomnia symptoms, and depressive symptoms, which were selected by univariable linear regression models with *P *<0.15 (See Table S3). Backward selection was used with retention at *P *<0.10. Age and insomnia symptoms were enforced and included in the final model where appropriate. Only variables included in final model were presented.

b When both insomnia and depressive symptoms were simultaneously included in the model, they remained insignificant (*P*=0.730, and *P*=0.116, respectively). CI, confidence interval; WHR, waist to hip circumference ratio.

### Sensitivity Analysis

Sensitivity analysis was performed with insomnia symptoms treated as a continuous variable. There was a significant interaction between age group and insomnia symptoms as a continuous variable on LTL (-3.9%, 95% CI -6.9% to -0.8%, *P*_interaction_=0.013) in all participants after adjusting for HIV serostatus, gender, education, smoking history, and depressive symptoms. Repeating the analyses in each group showed that this interaction remained significant among HIV-infected participants (-6.1%, 95% CI -10.1 to -1.9%, *P*_interaction_=0.005), which was not the case among HIV-uninfected participants (-1.1%, 95% CI -5.5% to 3.5%, *P*_interaction _=0.632). Of note, among the old HIV-infected participants, when insomnia symptoms as a continuous variable and depressive symptoms were simultaneously adjusted, the former was significantly associated with shorter LTL (-4.5%, 95% CI -8.2% to -0.8%, *P*=0.019) but the latter was not significant (*P*=0.403).

## DISCUSSION

We observed that HIV infection was associated with shorter LTL with a marginal significance after controlling for potential confounders, consistent with previous studies [19,20]. However, this association was markedly attenuated after additionally controlling for depressive and insomnia symptoms, suggesting that insomnia and depressive symptoms may be important factors contributing to the observed association between HIV infection and shorter LTL. This is supported by our previous observations in the same study population in which higher prevalence of frailty in HIV-infected adults compared with HIV-uninfected adults is partly attributed to higher level of neurocognitive impairment and depressive symptoms in HIV-infected adults [27]. Telomere shortening is a hallmark of cellular senescence [8], the accumulation of cellular stress will initiate cell cycle arrest and promote cellular senescence [36]. Studies indicated that sleep deprivation increases cellular stress, since sleep is considered to be a restorative process [4,5], and shorter leukocyte TL has been associated with sleep disturbance and insomnia [9-11]. The association between depression and accelerated aging at the cellular level has been reported in the general population [24]. Given that HIV-infected individuals experience elevated levels of depressive and insomnia symptoms [21,22], their negative effects on biological aging among HIV-infected individuals require more attention.

A notable finding was that that significant interaction of age group and insomnia symptoms on shorter LTL in HIV-infected patients was identified; specifically, insomnia symptoms were associated with shorter LTL in old HIV-infected individuals than but not those aged 40-54 years. Such findings have been previously reported in the general population [11,12]. However, we did not observe such interaction effect among HIV-uninfected individuals although similar unadjusted trend was observed. The discrepancy between our study and those of others might be partly explained by us having used a lower limit of old age group than earlier studies (55 vs 60 or 70 years) [11,12]. It is possible that the association of chronological age and insomnia symptoms on shorter LTL begins at an earlier age in adults living with HIV than in the general population, perhaps due to the reduced capacity of response to stress from aggregate effects of multiple comorbid factors, including HIV-related and treatment factors, coinfections, and other common risk factors [15,25,37]. Accumulating evidence indicates that insomnia leads to accelerated biological aging particularly among vulnerable population [12,38]. Prior research showed that frailty, an integrative marker of health and vulnerability, was more common among HIV-infected individuals than the HIV-uninfected [27,39].

We also found that similar to insomnia symptoms, depressive symptoms were independently associated with shorter LTL among old HIV-infected individuals, but none of them was significant when both were included in final model. This might be due to the fact that they are highly correlated, as our data and other studies demonstrated that depression was most strongly associated with insomnia in both HIV-infected and HIV-uninfected individuals [21,22]. But, it is notable that insomnia symptoms as a continuous variable was independently associated with shorter LTL among old HIV-infected individuals even adjusting for depressive symptoms, but the latter was not significant in this model. This suggests that insomnia may play a more direct effect on biological aging among old HIV-infected individuals, but requires further investigation using a longitudinal design.

Consistent with findings from an earlier study [20], we did not find associations between LTL, years since HIV diagnosis, antiretroviral treatment, and nadir CD4 levels. Zanet et al. reported that peak HIV RNA was associated with shorter LTL [20]. In our study, HIV-infected individuals on treatment with HIV RNA of <200 copies/mL had obvious longer LTL than those with HIV RNA of ≥200 copies/mL, even when adjusting for age, although it was not statistically significant (data notj shown). In Pathi et al. study [19], among HIV-infected patients with undetectable viral load, current CD4 count was positively associated with LTL, we found such association in all HIV-infected individuals. These suggests that HIV infection itself and viral load may not fully explain the accelerated biological aging observed in HIV-infected individuals.

Insomnia among HIV-infected individuals has been associated with antiretroviral drugs such as efavirenz, especially during the early period of receiving treatment [40]. But we didn’t find any HIV-specific factors independently associated with insomnia among HIV-infected individuals, only shorter duration of treatment was associated with insomnia symptoms in univariable analysis. In addition to depression mentioned before, the other significant variable associated with increased likelihood of insomnia symptoms was old age, but this association was identified only in HIV-uninfected but not HIV-infected individuals. This is consistent with a previous study of HIV-infected and -uninfected women [22]. An explanation is that old persons living with HIV tend to experience less psychological stress associated with HIV disease than younger HIV-infected individuals as a prior study reported [41].

There were some limitations to the current study. First, insomnia-related daytime dysfunctions were not assessed and precluded a formal insomnia diagnosis. In our study, a frequency of most nights or every night defined the existence of insomnia symptoms, such a stringent definition could lead to a measure of relatively severe insomnia symptoms, which may explain the lower prevalence of insomnia symptoms compared to studies using Pittsburgh Sleep Quality Index (43-66%) [21,26,42,43]; and also this measure detected a similar prevalence of insomnia symptoms in control sample as that estimated for the general population in China using DSM-IV (9.2%) [44]. Secondly, insomnia symptoms are usually concurrent with high level of depression, thereby making it difficult to detangle depression from insomnia and vice versa. Thirdly, the short duration of time since HIV diagnosis and treatment may limit our ability to investigate their effects on LTL. Fourthly, this study was a one-time measure of LTL, which might fluctuate over time. Additionally, the data were cross-sectional in nature, limiting the ability to draw causal inference.

The current study suggest that elevated insomnia and depressive symptoms may partly explain the association between HIV serostatus and shorter LTL. Besides, the identified significant association between insomnia and shorter LTL in old HIV-infected but not in uninfected individuals, suggest that such adverse effect may begin at an earlier age or is more pronounced in HIV-infected individuals. This may apply to persons with other chronic diseases associated with accelerated aging such as diabetes and cancers [37,38], but requires further investigation. Interventions aimed at improving sleep quality and mental health may help to lessen the adverse effects on cellular aging in HIV-infected individuals particularly those aged 55 or older.

## Supplementary data

Supplementary data is available online at www.aginganddisease.org/EN/10.14336/AD.2018.0204
