# Supplementary material for: Stronger Association between Insomnia Symptoms and Shorter Telomere Length in Old HIV-Infected Patients Compared with Uninfected Individuals
Source: Aging Dis. 2018 Dec 4;9(6):1010–9. doi: 10.14336/AD.2018.0204 (PMC6284770; doi:10.14336/AD.2018.0204)
Supplement: Supplementary file 1 — Supplementary data is available online at www.aginganddisease.org/EN/10.14336/AD.2018.0204 [file AD-9-6-1010-s.pdf]

SUPPLEMENTARY DATA

Stronger Association between Insomnia Symptoms and Shorter Telomere Length in Old HIV-Infected Patients Compared with Uninfected Individuals

Yingying Ding[1,2], Haijiang Lin[3], Sujuan Zhou[1,2], Keran Wang[1,2], Lingling Li[1,2], Yucheng Zhang[1,2], Yuan Yao[1,2], Meiyang Gao[1,2], Xing Liu[1,2], Na He[1,2, *]

[1] Department of Epidemiology, School of Public Health, Fudan University, Shanghai, China
[2] The Key Laboratory of Public Health Safety of Ministry of Education, Fudan University, Shanghai, China
[3] Taizhou City Center for Disease Control and Prevention, Taizhou City, Zhejiang Province, China

© 2018. Ding Y et al. Published online at http://www.aginganddisease.org/EN/10.14336/AD.2018.0204

SUPPLEMENTARY DATA

Supplementary Table 1. Univariable and multivariable Logistic regression analyses of possible correlates of insomnia symptoms in HIV-infected and HIV-uninfected participants.

	HIV-infected participants				HIV-uninfected participants			
	cOR (95% CI)	*P*	aOR (95% CI)[a]	*P*	cOR (95% CI)	*P*	aOR (95% CI)[a]	*P*
Age, years								
40-54	1.00				1.00			
55-82	1.20 (0.62–2.33)	0.585			1.75 (0.74–4.15)	0.205		
Age as a continuous variable	0.99 (0.96–1.03)	0.682	0.99 (0.96–1.04)	0.880	**1.05 (1.01–1.10)**	**0.014**	1.04 (0.99–1.09)	0.057
Male	0.68 (0.34–1.36)	0.276			0.51 (0.21–1.26)	0.145	0.44 (0.17–1.14)	0.092
Junior Middle school or above	0.62 (0.33–1.19)	0.150			0.72 (0.30–1.71)	0.458		
BMI, kg/m^2	0.93 (0.84–1.03)	0.188			0.91 (0.81–1.02)	0.118		
WHR above the cutoff [b]	0.51 (0.27–0.98)	0.043			1.15 (0.49–2.70)	0.745		
Smoking status								
Previous smoker (vs never)	0.27 (0.06–1.21)	0.087			1.22 (0.25–5.95)	0.802		
Current smoker (vs never)	0.84 (0.41–1.73)	0.633			0.69 (0.27–1.78)	0.442		
Current alcohol user [c]	1.25 (0.39–4.00)	0.704						
Depressive symptoms as continuous variable	**1.28 (1.18–1.40)**	**<.001**	**1.28 (1.18–1.40)**	**<.001**	**1.20 (1.08–1.32)**	**<.001**	**1.19 (1.07–1.32)**	**0.001**
HIV-related characteristics								
Homosexual HIV transmission	1.09 (0.47–2.53)	0.838						
Years since HIV diagnosis	0.87 (0.74–1.01)	0.062						
Nadir CD4 count (cells/µL)								
100–199 (vs <100)	1.40 (0.60–3.26)	0.440						
⩾ 200 (vs < 100)	1.06 (0.45–2.50)	0.890						
Current CD4 count ⩾ 200 cells/µL	0.73 (0.32–1.68)	0.463						
Using cART at enrollment								
cART naive	0.79 (0.25–2.53)	0.690						
on cART < 3 years	1.00							
on cART ⩾ 3 years	0.39 (0.18–0.84)	0.016						
Using EFV at enrollment	0.86 (0.45–1.63)	0.640						
Plasma HIV RNA < 200 copies/mL [d]	1.25 (0.26–5.94)	0.779						

[a]Variables with *P* <0.15 in univariate analysis were used in the development of a multivariate model with enforced entry of age. Backward selection was used with retention at *P* <0.10.
[b]Values of ≥0.9 for men and ≥0.85 for women define abdominal obesity.
[c]None of HIV-uninfected participants with insomnia symtoms were current alchol user (0/23).
[d]Data are available for 175 HIV-infected participants.
aOR, adjusted odds ratio; BMI, body mass index; cART, combination antiretroviral therapy; CI, confidence interval; cOR, crude odds ratio; WHR, waist to hip circumference ratio.

© 2018. Ding Y et al. Published online at http://www.aginganddisease.org/EN/10.14336/AD.2018.0204

SUPPLEMENTARY DATA

Supplementary Table 2. Sample characteristics of HIV-infected participants by HIV RNA level.

	HIV RNA < 200 copies/mL	HIV RNA ⩾ 200 copies/mL	With no available HIV RNA data close to survey	*P*[a]
Subjects	158	17	69	
Age, years	52.1±9.0	55.2±10.7	52.1±9.0	0.548
40-54	102 (64.6)	8 (47.1)	47 (68.1)	
55-82	56 (35.4)	9 (52.9)	22 (31.9)	
Male	113 (71.5)	13 (76.5)	54 (78.3)	0.550
Junior middle school or above	95 (60.1)	10 (58.8)	40 (58.0)	0.954
BMI, kg/m^2	21.8 (20.3–23.8)	22.6 (20.0–24.0)	22.3 (20.7–24.1)	0.565
Waist circumference, cm	80.0 (75.0–86.0)	85.0 (78.0–87.0)	82.0 (78.0–88.0)	0.190
Hip circumference, cm	90.0 (85.0–95.0)	96.0 (84.0–97.8)	90.0 (85.0–96.0)	0.594
WHR above the cutoff[b]	98 (62.0)	9 (52.9)	47 (68.1)	0.303
Smoking status				0.953
Current smoker	94 (59.5)	9 (52.9)	42 (60.9)	
Previous smoker	18 (11.4)	2 (111.8)	9 (13.0)	
Never smoker	46 (29.1)	6 (35.3)	18 (26.1)	
Current alcohol user	10 (6.3)	1 (5.9)	7 (10.1)	0.582
Depressive symptoms as a continuous variable	16.1±4.9	16.9±5.0	17.5±4.7	0.137
Insomnia symptoms	28 (17.7)	4 (23.5)	14 (20.3)	0.792
Insomnia symptoms as a continuous variable	6.4±2.6	7.1±3.0	6.7±2.8	0.608
HIV-related parameters				
Homosexual HIV transmission	28 (17.7)	3 (17.7)	14 (20.3)	0.504
Years since HIV diagnosis	3.6 (2.0–5.3)	4.1 (2.1–6.6)	1.2 (0.6–3.0)	<.001
Nadir CD4 count, cells/µL				<.001
<100	46 (29.1)	4 (29.4)	9 (13.0)	
100–199	65 (41.1)	5 (41.2)	15 (21.7)	
⩾ 200	47 (29.8)	5 (29.4)	45(65.2)	
Current CD4 count ⩾ 200 cells/µL	135 (85.4)	13 (76.5)	57 (82.6)	0.588
Using cART at enrollment				<.001
cART naive	0 (0.0)	0 (0.0)	20 (29.0)	
On cART < 3 years	79 (50.0)	8 (47.1)	46 (66.7)	
On cART ⩾ 3 years	79 (50.0)	9 (52.9)	3 (4.3)	
Duration on cART, years[c]	3.0 (1.8–3.8)	3.0 (2.1–4.0)	0.3 (0.2–0.6)	<.001
Using EFV at enrollment	78 (49.4)	8 (47.1)	33 (47.8)	0.967
Leukocyte telomere length (LTL)	0.9±0.4	0.8±0.3	1.0±0.5	0.026
Geometrc mean LTL[e]	0.80 (0.74–0.86)	0.72 (0.58–0.89)	0.90 (0.80–1.02)	0.075

Data are no. (%), mean±SD or median (interquartile range [IQR]), unless otherwise indicated.
[a] By the χ^2, Fisher's exact test or Anova, Kruskal-Wallis, as appropriate.
[b] Values of ≥0.9 for men and ≥0.85 for women define abdominal obesity.
[c] Data are available for 224 HIV-infected cART recipients.
[d] Data are available for 175 HIV-infected participants.
[e] Refers to geometric mean and 95% confidence interval.
BMI, body mass index; cART, combination antiretroviral therapy; WHR, waist to hip circumference ratio.

© 2018. Ding Y et al. Published online at http://www.aginganddisease.org/EN/10.14336/AD.2018.0204

SUPPLEMENTARY DATA

Supplementary Table 3. Univariable analyses of the association between various factors and leukocyte telomere length in all participants and separated by HIV serostatus and age group.

	All participants		Age 40-54 y		Age 55-82 y	
	HIV+	HIV-	HIV+	HIV-	HIV+	HIV-
	Percentage change (95% CI)	Percentage change (95% CI)	Percentage change (95% CI)	Percentage change (95% CI)	Percentage change (95% CI)	Percentage change (95% CI)
Age as a continuous variable	-0.4 (-1.0 to 0.3)	**-0.5 (-1.0 to 0.1)**[†]	-0.6 (-2.6 to 1.4)	**-1.6 (-3.3 to 0.1)**[†]	-0.8 (-2.5 to 0.8)	**-1.0 (-2.4 to 0.3)**[‡]
Male	-2.2 (-14.4 to 11.7)	**-9.2 (-19.0 to 1.8)**[†]	-9.6 (-23.5 to 6.9)	-7.5 (-19.8 to 6.7)	10.7 (-11.0 to 37.7)	-12.3 (-27.5 to 6.1)
Junior middle school or above	6.1 (-5.8 to 19.4)	**-9.8 (-18.6 to -0.2)**[*]	4.3 (-11.5 to 22.9)	-5.0 (-17.4 to 9.2)	7.1 (-13.0 to 31.9)	**-23.8 (-36.0 to -9.2)**[**]
BMI, kg/m^2	-0.6 (-2.3 to 1.2)	-0.1 (1.5 to 1.3)	-1.2 (-3.2 to 0.8)	0.1 (-1.6 to 1.8)	1.3 (-2.4 to 5.2)	-0.8 (-3.3 to 1.8)
WHR above the cutoff[a]	-1.7 (-12.9 to 10.9)	-4.4 (-13.6 to 5.8)	-0.4 (-14.2 to 15.6)	3.2 (-8.9 to 16.8)	-3.7 (-21.9 to 18.9)	**-16.7 (-30.5 to -0.2)**[†]
Smoking status						
Previous smoker (vs never)	-6.2 (-22.1 to 12.9)	-4.6 (-22.6 to 17.4)	-15.1 (-34.5 to 10.2)	-12.1 (-35.1 to 18.9)	6.1 (-19.2 to 39.3)	6.3 (-21.3 to 43.6)
Current smoker (vs never)	-0.1 (-85.7 to 596.1)	-6.4 (-15.8 to 4.1)	-4.2 (-18.4 to 12.4)	**-10.5 (-21.4 to 1.8)**[†]	8.4 (-14.8 to 37.9)	2.5 (-14.9 to 23.5)
Current alcohol user	11.5 (-10.8 to 39.3)	5.7 (-12.0 to 27.0)	0.3 (-24.5 to 33.3)	1.5 (-22.2 to 32.6)	**32.3 (-7.7 to 89.5)**[‡]	11.4 (-14.2 to 44.7)
Depressive symptoms as a continuous variable	-0.5 (-1.7 to 0.7)	-2.5 (-3.8 to -1.2)[***]	0.5 (-1.0 to 2.0)	**-2.3 (-3.9 to -0.6)**[**]	**-2.4 (-4.4 to -0.4)**[*]	**-2.8 (-5.1 to -0.6)**[*]
Insomnia symptoms	-5.3 (-18.4 to 10.0)	-8.1 (-22.7 to 9.1)	6.8 (-11.7 to 29.0)	2.0 (-19.2 to 28.9)	-21.6 (-38.3 to -0.3)[*]	-17.8 (-36.6 to 6.5)
Insomnia symptoms as a continuous variable	**-1.9 (-4.0 to 0.2)**[†]	**-2.9 (-5.1 to -0.7)**[*]	0.4 (-2.3 to 3.2)	**-2.4 (-5.5 to 0.8)**[‡]	**-5.3 (-8.4 to -2.2)**[**]	**-3.2 (-6.5 to 0.1)**[†]
HIV-related variables						
Homosexual HIV transmission	5.0 (-9.6 to 22.1)		4.9 (-11.7 to 24.7)		2.5 (-26.0 to 42.0)	
Years since HIV diagnosis	1.2 (-1.2 to 3.7)		0.5 (-2.5 to 3.5)		2.7 (-1.4 to 7.0)	
Nadir CD4 count (cells/μL)						
100–199 (vs < 100)	9.7 (-5.9 to 27.8)		**14.8 (-4.6 to 38.2)**[‡]		1.0 (-23.5 to 33.3)	
≥ 200 (vs < 100)	6.6 (-8.2 to 23.8)		9.1 (-9.0 to 30.8)		1.8 (-22.5 to 33.7)	
Current CD4 count ≥ 200 cells/μL	**14.4 (-2.3 to 34.1)**[†]		**17.6 (-3.4 to 43.2)**[‡]		8.9 (-16.8 to 42.5)	
Using cART at enrollment						
cART naïve vs on ART < 3 years	13.2 (-9.0 to 40.9)		**25.1 (-2.2 to 60.2)**[†]		-24.6 (-53.3 to 21.7)	
on cART ≥ 3 vs < 3 years	2.3 (-9.6 to 15.8)		1.7 (-12.8 to 18.6)		3.4 (-16.0 to 27.3)	
Using EFV at enrollment	-0.03 (-12.6 to 14.3)		2.2 (-13.6 to 21.0)		-3.5 (-22.8 to 20.7)	
Plasma HIV RNA < 200 copies/mL[b]	11.3 (-10.2 to 39.4)		-4.9 (-36.5 to 42.5)		**33.8 (-2.4 to 83.5)**[†]	

[‡] P <0.15, [†] P <0.1, [*] P <0.05, [**] P <.01, [***] P <.001.

[a] Values of ≥0.9 for men and ≥0.85 for women define abdominal obesity.

[b] Data are available for 175 HIV-infected participants.

BMI, body mass index; cART, combination antiretroviral therapy; CI, confidence interval; WHR, waist to hip circumference ratio.

© 2018. Ding Y et al. Published online at http://www.aginganddisease.org/EN/10.14336/AD.2018.0204
